# High-performance activated carbon from coconut shells for dye removal: study of isotherm and thermodynamics†

**DOI:** 10.1039/d4ra06287f

**Published:** 2024-10-24

**Authors:** Junaid Saleem, Zubair Khalid Baig Moghal, Snigdhendubala Pradhan, Gordon McKay

**Affiliations:** a Division of Sustainable Development, College of Science and Engineering, Hamad Bin Khalifa University, Qatar Foundation Doha Qatar jsaleem@hbku.edu.qa; b Center for Advanced Materials, Qatar University Qatar zubairkhalid009@gmail.com

## Abstract

This study investigates the production of high-performance activated carbon (AC) from coconut shells (CS) through acid and base activation processes, along with pre- and post-functionalization of the biochar, aiming to effectively remove dyes from aqueous solutions. The resulting AC exhibited outstanding adsorption capabilities, with the Langmuir model providing a good fit to the experimental data. Maximum adsorption capacities were observed at different temperatures: 805 mg g^−1^ at 298 K, 904 mg g^−1^ at 318 K, and 1000 mg g^−1^ at 338 K for NaOH-activated AC, and 252 mg g^−1^ at 298 K, 295 mg g^−1^ at 318 K, and 305 mg g^−1^ at 338 K for H_2_SO_4_-activated AC. The presence of active sites and functional groups on the surface of AC facilitated dye adsorption. The influence of various parameters, including adsorbent dosage, dye concentration, pH, and temperature, on the adsorption process were also examined, identifying the ideal conditions for dye removal. Thermodynamic analysis confirmed the endothermic nature of the adsorption process, with higher temperatures leading to increased adsorption capacities. Overall, the research highlights the potential of various activation routes for the production of high-value AC as a sustainable and effective adsorbent for dye removal from wastewater.

## Abstract 2

This study explores producing high-performance activated carbon from coconut shells using acid and base activation, along with biochar functionalization, to efficiently remove dyes from water.

## Graphical abstract

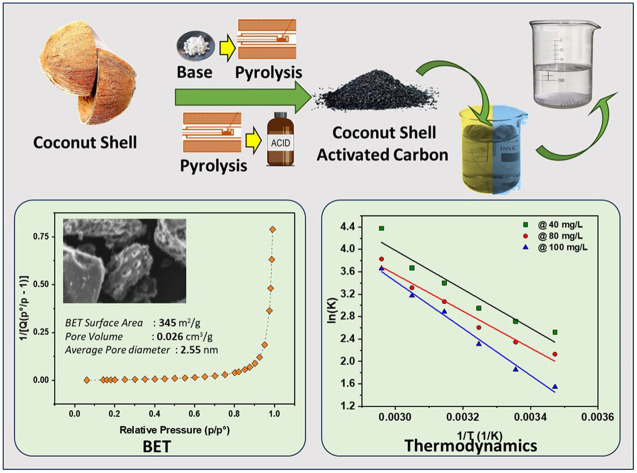

## 1 Introduction

The interest in the production of activated carbon (AC) from biomass represents a significant shift towards sustainable materials engineering.^1,2^ This innovative approach not only reduces energy consumption and minimizes carbon emissions but also aligns with the global push for sustainable development. The exceptional surface area and conductivity of AC make it a versatile material, crucial for effectively removing organic compounds, pollutants, and dyes from water, and thus an indispensable tool in water purification.^3,4^ Furthermore, the utilization of AC as an electrode material in supercapacitors, fuel cells, and batteries demonstrates its multifaceted role in the advancement of energy storage technologies.^5,6^ This dual functionality of AC, coupled with its sustainable production methods, highlights the material's potential to contribute significantly to both environmental protection and the development of renewable energy sources.

Despite the successful conversion of various biomass wastes into AC,^7–11^ coconut shells (CS) have emerged as a particularly advantageous feedstock due to the substantial waste generated by the coconut industry, which can be transformed into high-quality adsorbents.^12^ The effectiveness of CS in producing ACs with high surface areas makes them exceptionally suitable for applications such as wastewater treatment, drug delivery systems, and energy storage devices.^13^ This versatility is especially crucial in addressing the growing environmental and health concerns associated with the discharge of organic dyes into water bodies.^14^ These dyes can cause severe biological damage to aquatic life and pose significant health risks to humans through various forms of exposure. High-quality AC derived from CS offers a promising solution, combining effective dye removal with sustainable waste management and pollution control strategies, thereby safeguarding both environmental integrity and public health.^15^

In the context of utilizing CS as a feedstock for AC production, several studies have been carried out thus.^16–25^ A critical aspect highlighted in one study is the importance of thoroughly understanding the physicochemical properties of CS biomass prior to its thermo-chemical conversion. This knowledge is essential for producing high-grade charcoal and for tailoring the conversion treatments to minimize greenhouse gas emissions, thereby enhancing the sustainability of the process.^26^ Also, biochar from CS effectively remediated three reactive dyes, achieving a maximum adsorption of 73.03 mg g^−1^.^27^ Elsewhere, the pore properties and textural characterization of AC derived from dried CS were investigated using an environment-friendly CO_2_ activation process.^28^ In one study, CS-based AC effectively removed metal ions, showing a maximum adsorption capacity of 26.50 mg g^−1^ specifically for lead from aqueous solutions.^29^ Moreover, mesoporous AC was prepared using a hydrochar derived from CS waste through hydrothermal carbonization and NaOH chemical activation process.^30^ Recently, doped porous biochar using CS has also been reported. For instance, boron-doped porous biochar was synthesized *via* a microwave-assisted pyrolysis method using boric acid as a dopant and CS powder as a carbon source for efficient removal of aqueous tetracycline antibiotics.^31^ Another study developed a catalytic CO_2_ absorption process using CS-derived nitrogen-doped biochar (NBC), achieved through urea modification and KOH activation, to enhance CO_2_ capture in an amine-based solution.^32^ These findings demonstrate the potential of CS as a superior and more environmentally friendly feedstock for AC production, contributing to the development of sustainable waste management and pollution control strategies.

To the best of our knowledge, no previous studies have simultaneously examined acid (H_2_SO_4_) and base (NaOH) activation processes along with pre- and post-functionalization of CS biochar, which yields exceptionally high dye adsorption capacity supported by detailed isotherm and thermodynamic studies. In this work, we produced AC using CS feedstock to explore combined activation and functionalization methods. We conducted a comprehensive study that includes morphology through SEM and BET surface area analysis, as well as chemical and structural characterization through XRD and FTIR.

## 2 Methodology

### 2.1. Dye preparation

Rhodamine 6G (R6G), a cationic dye with a molar mass of 479.02 g mol^−1^ and Indigo Carmine (IC), an anionic dye with a molar mass of 466.36 g mol^−1^ were supplied by Sigma-Aldrich, USA and were utilized in the dye removal experiment. Different concentrations were prepared using deionized water.

### 2.2. AC preparation

Ten dried CS were purchased from a local supermarket and were used in the preparation of AC. Initially, the CS was cleaned with water to get rid of any impurities such as dirt or residual pulp. They were then cut into small pieces of 4–5 mm and then were dried under the sun for two days.

#### 2.2.1 Activation before pyrolysis

The dried CS pieces were dipped in 2 N NaOH and 2 M H_2_SO_4_ solution separately and then left to stir continuously throughout the night. After being soaked in the alkaline and acidic solutions, the CS pieces were rinsed with water to neutralize them and then dried in the shade. They were then encased in aluminium foil and placed inside a furnace, which was set to increase the temperature to 600 °C at a rate of 10 °C min, under nitrogen. The CS pieces were heated for 2 h at this temperature, after which they were allowed to cool down to room temperature, also at a rate of 10 °C min.

#### 2.2.2 Functionalization after pyrolysis

The dried CS pieces were pyrolyzed as described above. After pyrolysis, the biochar was dipped in 2 N NaOH and 2 M H_2_SO_4_ solution separately and then left to stir continuously throughout the night. After being soaked in the alkaline and acidic solutions, the CS pieces were rinsed with water to neutralize them and then dried in the shade. The Brunauer–Emmett–Teller (BET) and scanning electron microscopy (SEM) analysis were carried out to investigate the surface morphology and internal porous structure. The functional groups and chemical structure were analyzed by FTIR and XRD analysis.

### 2.3. Equilibrium study

100 ml of R6G with initial concentrations of 5–3200 mg L^−1^ and IC solutions with initial concentrations of 5–1200 mg L^−1^ were placed in the flasks. An equal mass of 0.1 g of the AC was added to each flask and kept in the shaker at 298 K for 24 h to reach equilibrium. The initial pH of the solutions, which was approximately 7, was maintained for the experiments. The same procedures were repeated for two additional sets of flasks, each containing the same starting concentrations of dye and the same quantity of AC, but these sets were maintained at different temperatures: 318 K and 338 K. Samples of the aqueous solutions were collected, and their concentrations were measured. Before analysis, all samples were centrifuged to reduce the interference of fine carbon particles with the measurements. Each experiment was conducted twice under the same conditions to ensure reliability. The concentrations of R6G and IC in the solutions were measured *via* a UV spectrophotometer. The adsorption amount at equilibrium, denoted as *Q*_e_ (mg g^−1^), was determined using the formula:*Q*_e_ = ((*C*_0_ − *C*_e_)*V*)/*W*In this equation, *C*_0_ and *C*_e_ (mg L^−1^) represent the initial and equilibrium concentrations of the dye in the liquid phase, respectively. *V* is the volume of the solution and *W* is the mass of the dry AC.

## 3 Results and discussion

### 3.1. Optimization of activation routes

The optimization of AC production for dye removal was investigated by comparing acid (H_2_SO_4_) and base (NaOH) activation methods, both before and after pyrolysis. The adsorption capacities of the AC were evaluated at different temperatures (298 K, 318 K, and 338 K) to determine the most effective activation route for both cationic and anionic dyes (Table 1).

**Table 1:** Adsorption capacities of CS through different routes for both acid *vs.* base activation

Temperature (K)	Adsorption capacity (mg g^−1^)			
	H_2_SO_4_ activation		NaOH activation	
	Before pyrolysis	After pyrolysis	Before pyrolysis	After pyrolysis
298	135	226	751	342
318	180	280	859	375
338	210	306	968	478

#### 3.1.1. H_2_SO_4_ activation

The results demonstrated that H_2_SO_4_ activation of AC, especially after pyrolysis, significantly improved the adsorption capacity for the anionic dye IC. Before pyrolysis, the adsorption capacities were 135 mg g^−1^, 180 mg g^−1^, and 210 mg g^−1^ at 298 K, 318 K, and 338 K, respectively. After pyrolysis, these values increased to 226 mg g^−1^, 280 mg g^−1^, and 306 mg g^−1^ at the same respective temperatures. The introduction of sulfonic groups during acid activation enhances the AC's affinity for anionic dyes. Therefore, H_2_SO_4_ activation post-pyrolysis was identified as the optimal route for maximizing the adsorption capacity of anionic dyes and was utilized as the default route for subsequent characterizations and results.

#### 3.1.2. NaOH activation

In contrast, NaOH activation prior to pyrolysis exhibited exceptionally high adsorption capacities for the cationic dye R6G. Before pyrolysis, the adsorption capacities were 751 mg g^−1^, 859 mg g^−1^, and 968 mg g^−1^ at 298 K, 318 K, and 338 K, respectively. After pyrolysis, these values were lower, at 342 mg g^−1^, 375 mg g^−1^, and 478 mg g^−1^ at the same respective temperatures. The creation of basic sites during NaOH activation facilitates the adsorption of cationic dyes. The lower capacities observed post-pyrolysis indicate that pre-pyrolysis activation is more effective for cationic dye removal. Thus, NaOH activation before pyrolysis was selected as the optimal route for achieving high adsorption capacities for cationic dyes and was used as the default route for subsequent characterizations and results.

### 3.2. Surface properties and morphology

**Fig. 1:**
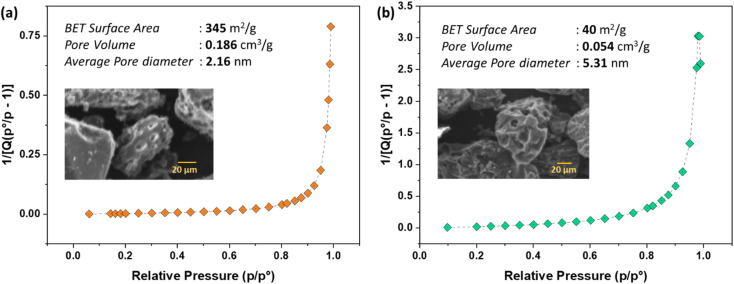
BET surface area plot with an SEM inset: for (a) NaOH, and (b) H_2_SO_4_ activation routes.

The BET surface area measurements provide insights into the textural properties of the AC (Fig. 1). For NaOH activation, the surface area was determined to be 345 m^2^ g^−1^, indicating a highly porous structure. The pore volume of 0.2 cm^3^ g^−1^ and an average pore size of 1.92 nm suggest that the AC has a significant amount of micropores (and mesopores). The insertion of sodium into the carbon material seemed to cause a significant increase in its volume, leading to the development of a substantial specific surface area.^33^ This high surface area and suitable pore volume are critical for adsorption processes as they allow for the efficient capture and storage of adsorbate molecules. These results are consistent with the previous studies reported in the literature.^28,30^ On the other hand, the BET surface area obtained using H_2_SO_4_ activation route was found to be 40 m^2^ g^−1^.

The combination of a high BET surface area and appropriate pore size distribution makes CS–AC an excellent candidate for applications requiring efficient adsorption. The microporous and mesoporous nature of the material enhances its capacity to adsorb small molecules, making it suitable for environmental remediation, gas storage, and purification processes.

Further, the SEM analysis of the porous AC adsorbent has provided valuable insights into the surface morphology, which is crucial for its performance in adsorption processes. The formation of such a porous structure is instrumental in enabling the efficient entry of dye-water into the matrix and subsequent adsorption, as evidenced by the presence of numerous active sites post-activation and pyrolysis, as shown in the inserts of Fig. 1. The results indicate that the adsorbent possesses the necessary characteristics for an effective adsorbent, including a high surface area, appropriate pore size, potentially favorable surface functional groups, and a large porous network that facilitates diffusion.^7^ These features are consistent with the observed performance in the adsorption of pollutants, as seen in the case of alizarin red S removal using sulfuric acid-modified avocado seeds, where the activating agent increased the surface area and pore density of the adsorbent, leading to improved adsorption efficiency.^34^

### 3.3. XRD and FTIR

**Fig. 2:**
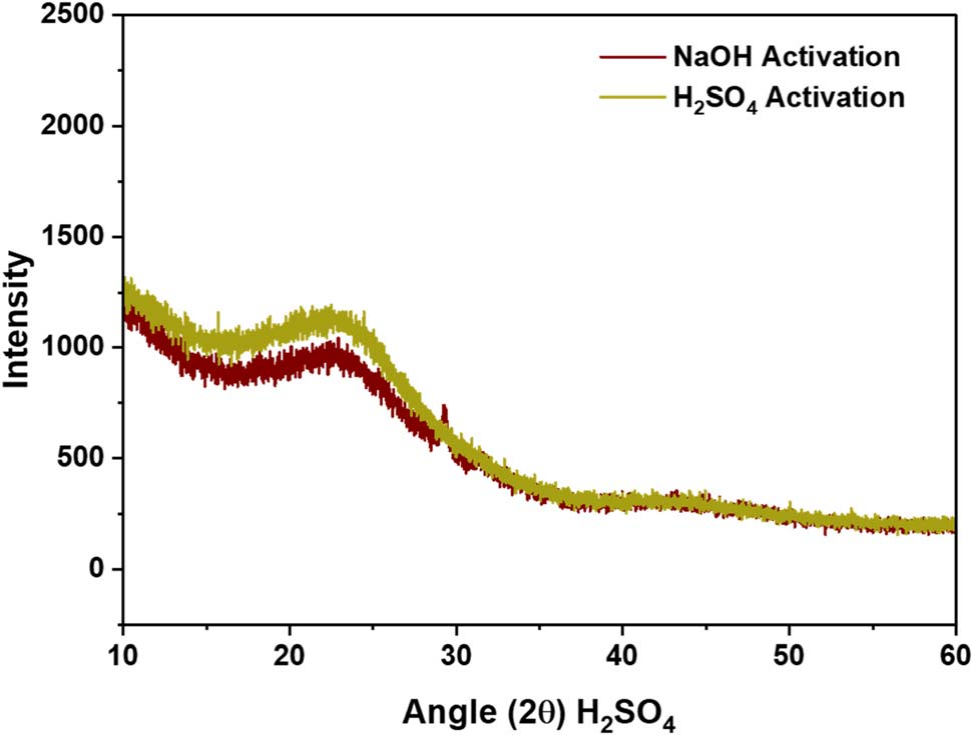
XRD spectra of CS-AC using NaOH and H_2_SO_4_ activation routes.

The XRD patterns, as depicted in Fig. 2, reveal identical spectra for AC synthesized *via* both activation routes. The broad diffraction bands, rather than distinct sharp peaks, suggest that the material is predominantly amorphous.^35,36^ Specifically, two characteristic peaks at 24° and 42° indicate the presence of graphite crystallites within the carbon scaffold. This disordered nature is advantageous as it provides a higher number of active sites and a larger surface area, thereby enhancing the adsorption properties of the AC.^34^

**Fig. 3:**
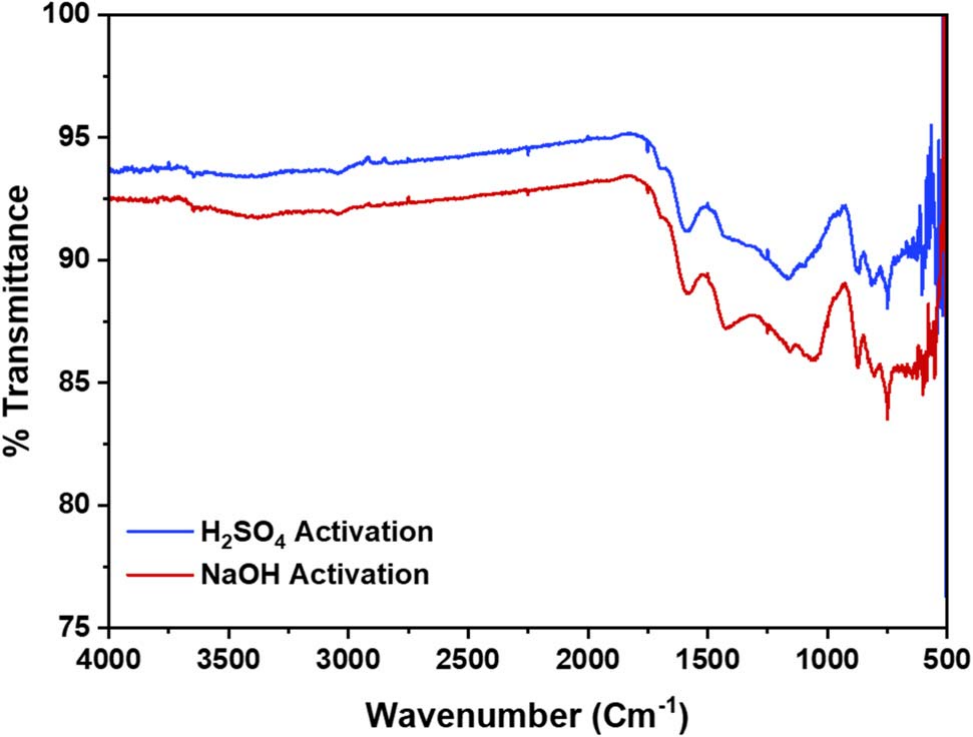
FTIR spectra of CS-AC using NaOH and H_2_SO_4_ activation routes.

Additionally, the presence of functional groups identified in the FTIR analysis further augments the material's adsorption potential by providing active sites for chemical interactions. The FTIR spectra further supports the structural analysis by revealing the chemical bonds present in the AC, Fig. 3. The spectra for both activation routes are similar, indicating that the activation method does not significantly alter the chemical structure. The range between 3300 and 3500 cm^−1^ is among the most commonly observed peaks in the spectra of ACs.^36^ These peaks are indicative of the stretching vibrations of O–H bonds in hydroxyl groups and N–H bonds in alkoxy groups. Additionally, sharp peak shifts at 1620–1625 cm^−1^ are indicative of C–O stretching in aldehyde and ketone groups.^34,37^ The broader peaks at 1160–1170 cm^−1^ indicate the existence of amine groups.^34^ These functional groups are essential as they contribute to the chemical reactivity and adsorption capacity of the AC.^33^

The XRD, FTIR, and BET analyses collectively demonstrate that the activated CS AC possesses a highly desirable combination of structural and textural properties. The amorphous nature, high surface area, significant microporosity, and presence of various functional groups all contribute to its effectiveness as an adsorbent material. This study underscores the potential of using CS as a sustainable and efficient precursor for producing high-quality AC for diverse adsorption applications.

### 3.4. Adsorption performance

**Fig. 4:**
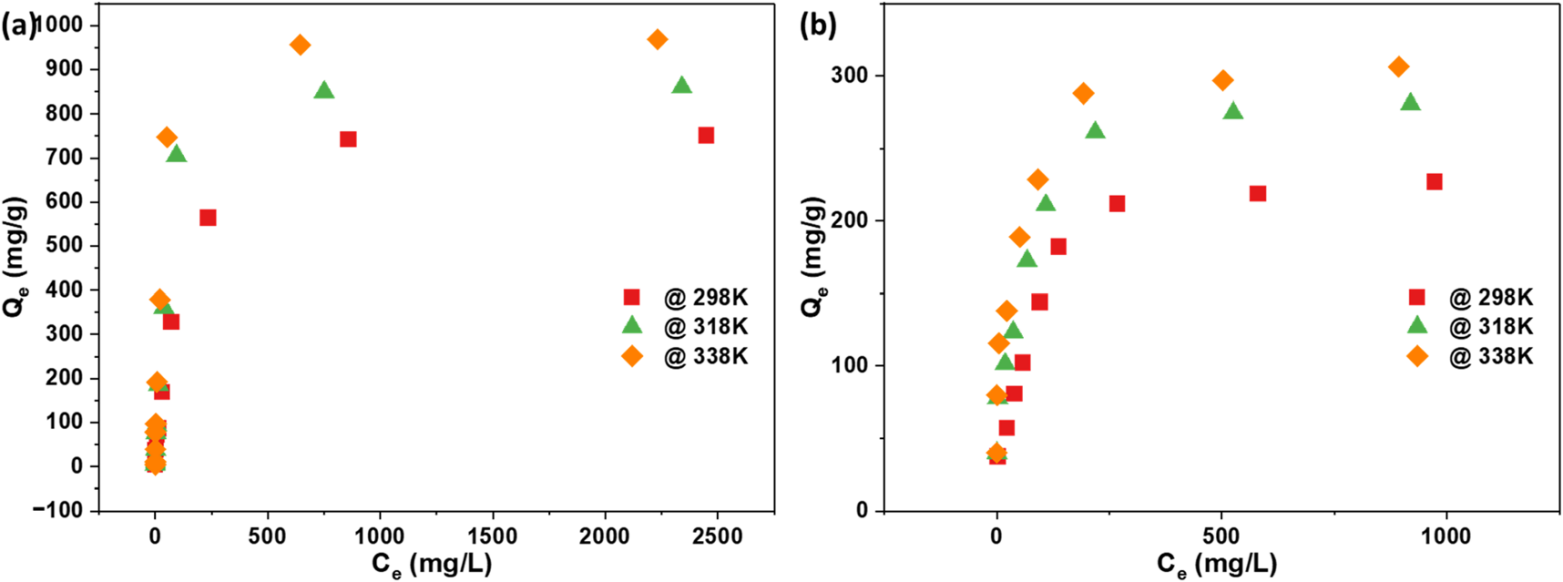
Adsorption isotherm for (a) NaOH; (b) H_2_SO_4_ activation routes at various temperatures.

Fig. 4 offers a detailed depiction of the adsorption isotherm for the synthesized AC. This isotherm illustrates the equilibrium relationship between the adsorbate concentration in the solution (*C*_e_) and the amount of adsorbate adsorbed per unit mass of the adsorbent (*Q*_e_). Understanding this relationship is crucial for analyzing the adsorption behavior and effectiveness of the AC in removing a particular adsorbate. From the data in Fig. 4, it's evident that the AC activated using NaOH shows a notable adsorption capacity of 751 mg g^−1^. In comparison, the AC activated with H_2_SO_4_ has a lower adsorption capacity of 226 mg g^−1^. This variation underscores the significant influence of the activation agent on the adsorption capabilities of the final AC product. Moreover, with increasing temperature, the adsorption capacity of the AC increases. Three temperatures were selected for both activation routes: 298 K, 318 K, and 338 K. At 338 K, the NaOH-activated AC achieved an adsorption capacity of 968 mg g^−1^, while the H_2_SO_4_-activated AC reached 306 mg g^−1^. This indicates that higher temperatures enhance the adsorption capacity, likely due to increased molecular movement and pore accessibility in the AC structure. These results highlight not only the importance of the activation agent but also the effect of temperature on the adsorption performance.

### 3.5. Adsorption isotherms

Understanding the equilibrium behavior of adsorbates on adsorbents is crucial for predicting and optimizing the performance of adsorption systems. The experimental data was compared with four widely recognized adsorption isotherm models: Langmuir, Freundlich, Temkin, and Redlich–Peterson. The Langmuir isotherm, which assumes monolayer adsorption onto a surface with a finite number of identical sites.^33^ The Freundlich isotherm, on the other hand, is empirical and describes heterogeneous surfaces and multilayer adsorption.^38^ The Temkin isotherm takes into account the interactions between adsorbents and adsorbates, assuming that the heat of adsorption decreases linearly with coverage.^39,40^ Lastly, the Redlich–Peterson model is often used to describe adsorption processes where the adsorbent exhibits heterogeneous surface properties. It is a hybrid isotherm that incorporates features of both the Langmuir and Freundlich isotherms.

To assess the applicability of these models to the adsorption study, a two-pronged strategy was employed. First, the proximity of the experimental results to the values obtained from the isotherm model was evaluated. This comparison provides an initial indication of which model best represents the experimental data. Second, the correlation coefficients (*R*^2^ values) were scrutinized. A higher *R*^2^ value indicates a better fit between the model and the experimental data.

**Fig. 5:**
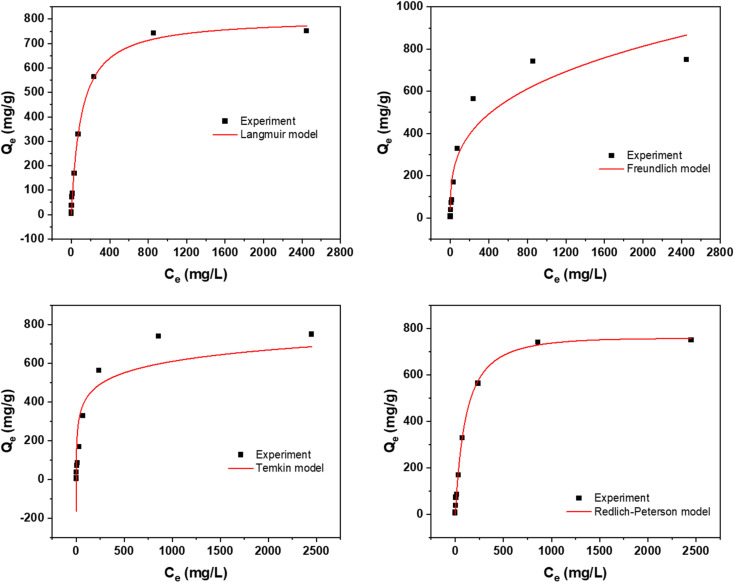
Isotherms at 298 K using NaOH activated CS in the removal of Rhodamine 6G dye.

**Fig. 6:**
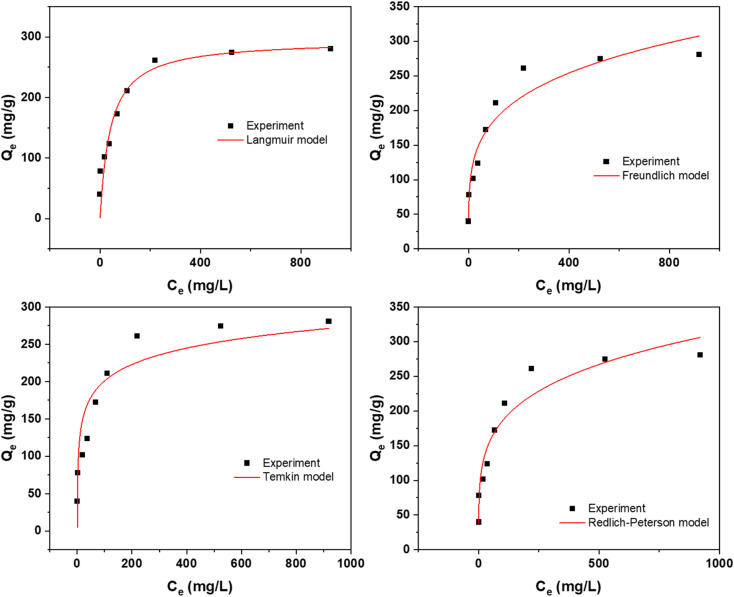
Isotherms at 298 K using H_2_SO_4_ activated CS in the removal of Indigo Carmine dye.

The results of this comprehensive isotherm study are presented in Fig. 5 and 6. Additionally, Tables 2 and 3 provide the model equations, parameters, and their corresponding values, allowing for a detailed analysis of the isotherm characteristics. The *R*^2^ value indicates that the adsorption data at all three temperatures studied adhered best to the Langmuir isotherm model.

**Table 2:** Various isotherms at 298 K using NaOH activation in the removal of Rhodamine 6G dye^a^

Model	Ind.	Dep.	Parameters	Values
Langmuir non-linear	*C*_e_	*Q*_e_	*q*_m_	804.523
			*K*_L_	0.00971
			*R* ^2^	0.99726
Freundlich non-linear	*C*_e_	*Q*_e_	*n*	3.20946
			*K*_F_	76.1631
			*R* ^2^	0.91263
Temkin	*C*_e_	*Q*_e_	*b*_T_	29.1327
			*A*_T_	1.30095
			*R* ^2^	0.83758
Redlich–Peterson	*C*_e_	*Q*_e_	*K*_R_	7.00377
			*a*_R_	0.00624
			*g*	1.04472
			*R* ^2^	0.99809

a Ind. = independent variable; Dep. dependent variable.

**Table 3:** Various isotherms at 298 K using H_2_SO_4_ activation in the removal of Indigo Carmine dye^a^

Model	Ind.	Dep.	Equation	Parameters	Values
Langmuir non-linear	*C*_e_	*Q*_e_	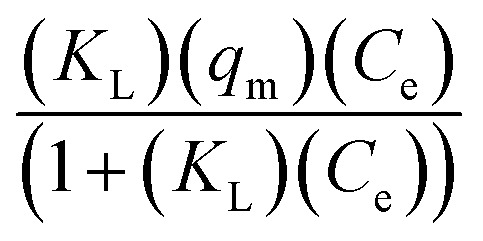	*q*_m_	252
				*K*_L_	0.01406
				*R* ^2^	0.96375
Freundlich non-linear	*C*_e_	*Q*_e_	(*K*_F_)(*C*_e_^1/*n*^)	n	3.49557
				*K*_F_	35.48788
				*R* ^2^	0.88103
Temkin	*C*_e_	*Q*_e_	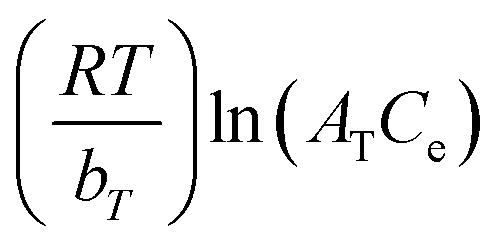	*b*_T_	64.60842
				*A*_T_	0.44599
				*R* ^2^	0.88634
Redlich–Peterson	*C*_e_	*Q*_e_	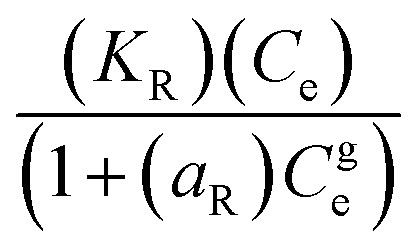	*K*_R_	2.6743
				*a*_R_	0.00386
				*g*	1.1516
				*R* ^2^	0.973

a Ind. = independent variable; Dep. dependent variable.

The study revealed a temperature-dependent increase in the maximum adsorption capacity, as predicted by the Langmuir model. For the NaOH activation route, the capacity was 805 mg g^−1^ at 298 K, which increased to 904 mg g^−1^ at 318 K and further to 1000 mg g^−1^ at 338 K. Similarly, for the H_2_SO_4_ activation route, the capacity was 252 mg g^−1^ at 298 K, rising to 295 mg g^−1^ at 318 K and reaching 305 mg g^−1^ at 338 K. The isotherms for 318 K and 338 K are provided in the ESI file, Fig S1–S4.†

### 3.6. Thermodynamic study

**Fig. 7:**
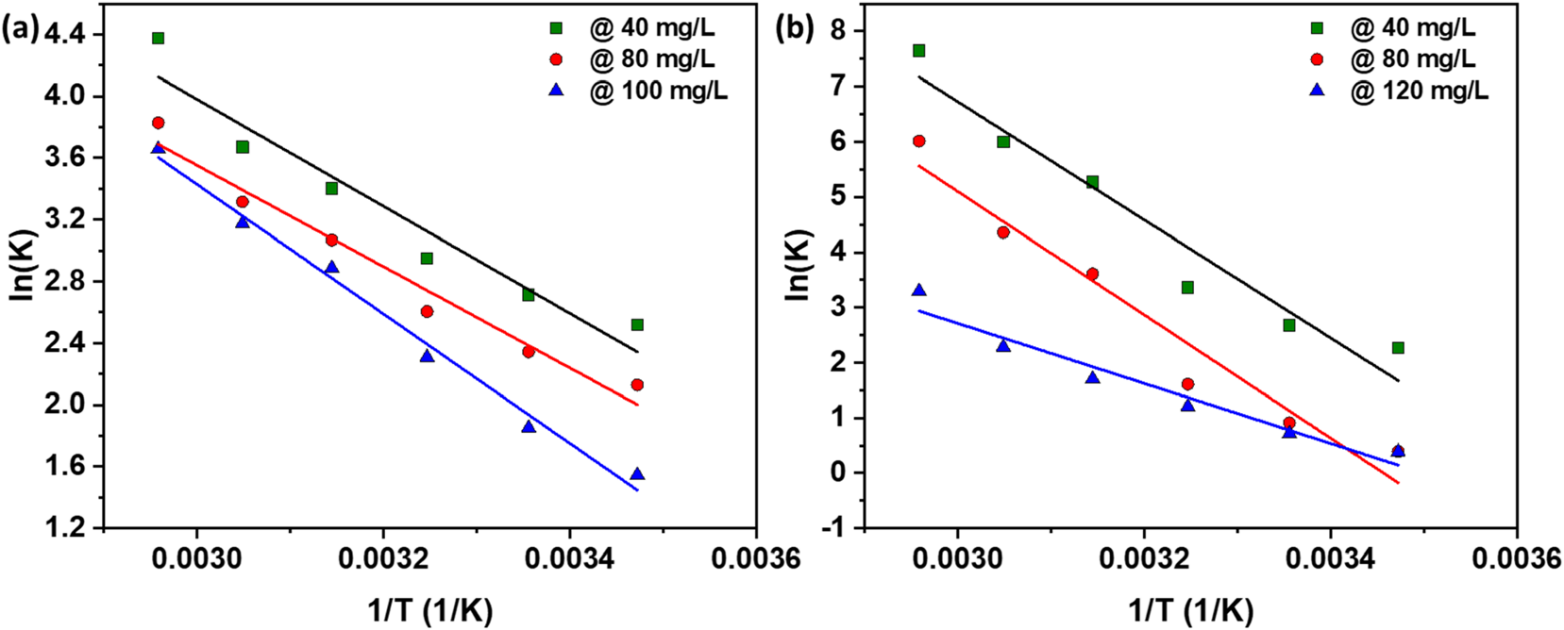
Thermodynamics study: plot of ln *K*_d_*vs.* 1/*T* at various initial dye concentrations using AC made from CS through (a) NaOH, and (b) H_2_SO_4_ activation routes.

The thermodynamic parameters Δ*H*°, Δ*S*°, and Δ*G*° calculated for the adsorption process are presented in Fig. 7 and summarized in Table 4 for NaOH and Table 5 for H_2_SO_4_. The positive values of Δ*H*° indicate that the adsorption interaction is endothermic. This conclusion is further supported by the data in Tables 2 and 3, where the maximum monolayer adsorption capacity increases from 805 to 1000 mg g^−1^ (for NaOH) and from 252 to 305 mg g^−1^ (for H_2_SO_4_) as the solution temperature rises from 298 K to 338 K. This increase in adsorption capacity with temperature demonstrates the endothermic nature of the process, confirming that higher temperatures facilitate greater adsorption. The positive value of Δ*S*° reveals the affinity of the AC for the dye, suggesting an increased disorder at the solid–solution interface during adsorption. This increase in randomness is characteristic of the adsorption process, indicating that the dye molecules are more freely arranged on the AC surface, thus enhancing the adsorption efficiency.^33^ Moreover, the negative value of Δ*G*° signifies the feasibility and spontaneous nature of the adsorption process.^33,36^ This negative Gibbs free energy change points to a high preference of the dye molecules for the AC, corroborating the spontaneous adsorption mechanism. This spontaneous nature is essential for practical applications, ensuring that the adsorption process occurs readily without requiring external energy input. These findings align with previous studies on the adsorption of various substances: adsorption of arsenic onto coconut husk,^41^ methylene blue (MB) onto oil palm fiber,^33^ MB onto corncorb,^42^ and anionic and cationic dyes onto rubber seed shell and rubber seed.^36^

**Table 4:** Thermodynamic values using AC through NaOH activation route

(K)	Δ*G*			Δ*H*			Δ*S*		
	40 mg L^−1^	80 mg L^−1^	120 mg L^−1^	40 mg L^−1^	80 mg L^−1^	120 mg L^−1^	40 mg L^−1^	80 mg L^−1^	120 mg L^−1^
288	−6031.67	−5095.91	−3699.7						
298	−6725.85	−5805.39	−4589.06	28 840.94	27 308.5	34 887.1	119.6205	111.465	133.1701
308	−7556.65	−6669.91	−5911.86						
318	−8988.87	−8112.34	−7636.11						
328	−10007.9	−9041.3	−8653.71						
338	−12296.5	−10759	−10282.1						

**Table 5:** Thermodynamic values using AC through H_2_SO_4_ activation route

(K)	Δ*G*			Δ*H*			Δ*S*		
	40 mg L^−1^	80 mg L^−1^	120 mg L^−1^	40 mg L^−1^	80 mg L^−1^	120 mg L^−1^	40 mg L^−1^	80 mg L^−1^	120 mg L^−1^
288	−5421.87	−937.648	−928.478						
298	−6639.34	−2259.14	−1786.04	88 954.6	92 845.82	45 192.13	322.7786	320.9295	158.1182
308	−8622.67	−4121.31	−3094.14						
318	−13927.4	−9546.73	−4507.1						
328	−16359.1	−11880.7	−6224.04						
338	−21495.3	−16899.3	−9261.74						

### 3.7. Adsorbent dosage

**Fig. 8:**
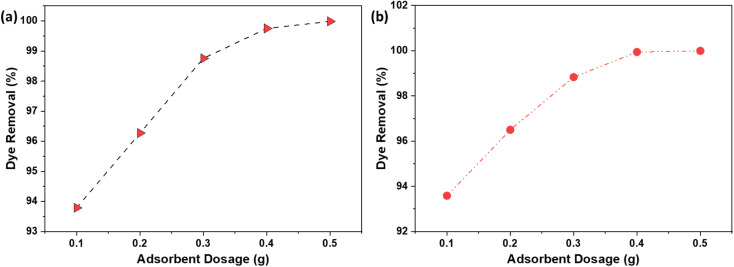
The effect of adsorbent dosage on the % dye removal using AC made from CS through (a) NaOH; (b) H_2_SO_4_ activation routes.

The optimization of adsorbent dosage is a critical parameter in the adsorption process, as it directly impacts the efficiency of dye removal and the economic viability of the treatment method. The results illustrated in Fig. 8a (NaOH) and 8b (H_2_SO_4_) provide valuable insights into the relationship between the dosage of AC and the removal % of the dye. As the AC dosage increased from 0.1 g to 0.5 g, the removal % of the dye improved from 93.7% to 99.9%. This enhancement can be attributed to the increased availability of active sites or surface area on the AC, which facilitates greater interaction between the dye molecules and the adsorbent.^36^ The additional active sites allow for more dye molecules to be adsorbed, thereby increasing the overall percentage of dye removed from the solution. Further increases in the adsorbent dosage led to a dye removal efficiency of 100%. At this point, the number of active sites available on the AC in the aqueous solution exceeded the number of the dye molecules present. Consequently, a surplus of active sites remained unutilized, indicating that the adsorbent dosage was more than sufficient for complete dye removal. By identifying the threshold dosage at which 100% dye removal is achieved, it is possible to avoid using excess adsorbent that does not contribute to improved performance. This not only reduces the amount of AC required but also minimizes the volume of spent adsorbent that must be disposed of, which is beneficial from both an economic and an environmental standpoint.

### 3.8. Dye concentration

**Fig. 9:**
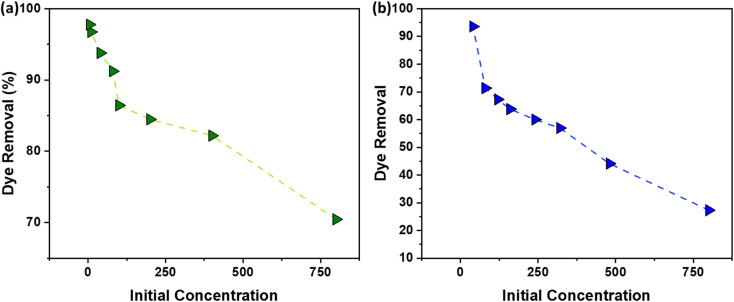
The effect of initial dye concentration on the % dye removal using AC made from CS through (a) NaOH; (b) H_2_SO_4_ activation routes.

The relationship between dye concentration (mg L^−1^) and the % of dye removal by AC adsorbent is a critical aspect of adsorption processes. As depicted in Fig. 9a (NaOH) and 9b (H_2_SO_4_), the removal efficiency of the dye decreases as the concentration of the dye increases. This phenomenon can be attributed to the saturation of available adsorption sites on the AC surface. When the number of dye molecules (pollutants) exceeds the number of active sites, the adsorption capacity of the AC becomes the limiting factor, leading to a decline in the uptake %.^36^ The data presented in Fig. 9a and b indicate that AC exhibits optimal uptake capacity at dye concentrations of 40 mg L^−1^ (ppm), regardless of whether NaOH or H_2_SO_4_ activation methods are employed. This suggests that the AC adsorbent has a specific number of active sites that are most effectively utilized when the dye concentration is at this level. At lower concentrations, the adsorbent may not be used to its full potential, while at higher concentrations, the adsorbent becomes saturated, and the efficiency of dye removal decreases.

### 3.9. pH

The pH of the solution plays a pivotal role in this interaction, as it influences both the surface charge of the adsorbent and the ionization state of the adsorbate. At lower pH levels, the higher concentration of H^+^ ions in the solution can lead to increased competition with the cationic dye molecules for adsorption sites, potentially reducing the adsorption efficiency. Conversely, as the pH increases and the concentration of H^+^ charges decreases, the competition between these positive ions and the dye molecules for adsorption sites diminishes. Moreover, at higher pH levels, the surface of the AC becomes more negatively charged due to the alkaline activation.^36^ This increases electrostatic interaction which contributes to a higher adsorption efficiency for the under alkaline conditions.

**Fig. 10:**
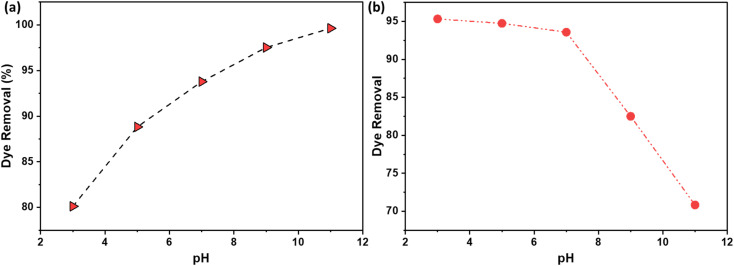
The effect of pH on the % dye removal using AC made from CS through (a) NaOH; (b) H_2_SO_4_ activation routes.

Fig. 10 illustrates the effect of pH on the percentage of dye removal using NaOH and H_2_SO_4_ activation routes. Dye adsorption onto activated carbon (AC) is a complex process influenced by factors such as the surface chemistry of the adsorbent, the chemical nature of the dye, and the pH of the solution. The activation method used to prepare AC significantly impacts its surface functional groups and, consequently, its adsorption properties.

For cationic dyes, which carry a positive charge, NaOH activation introduces alkaline surface functional groups onto the carbon. These groups interact favorably with the positively charged dye molecules through electrostatic attractions, resulting in enhanced adsorption, as shown in Fig. 10a. Conversely, for anionic dyes, which carry a negative charge, H_2_SO_4_ activation introduces acidic surface functional groups. These groups interact favorably with the negatively charged dye molecules, also through electrostatic attractions, leading to increased adsorption, as evidenced in Fig. 10b.

### 3.10. Temperature

**Fig. 11:**
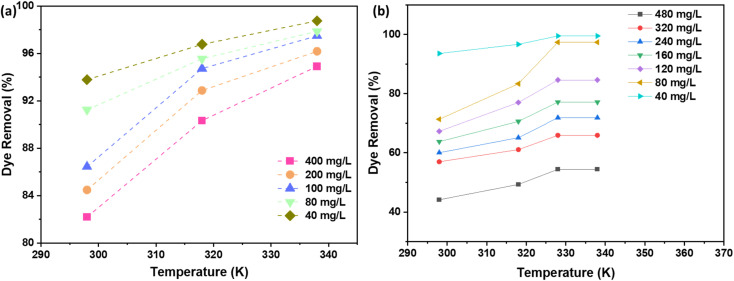
Temperature effect on the % dye removal using AC made from CS through (a) NaOH; (b) H_2_SO_4_ activation routes.

The influence of temperature on the adsorption process is a critical aspect to consider when designing efficient dye removal systems. In the present study, the effect of temperature on the removal % of the dye using AC through NaOH and H_2_SO_4_ activation routes was investigated. The results, as depicted in Fig. 11 indicate that the removal % of the dye increases with an increase in the solution temperature. It was attributed to the increase in the mobility of the dye molecules, which facilitates their diffusion from the bulk solution to the surface of the AC. This increased mobility allows for a more efficient interaction between the active sites on the AC and the dye molecules, resulting in a higher removal %.

### 3.11. Repeatability experiments

**Fig. 12:**
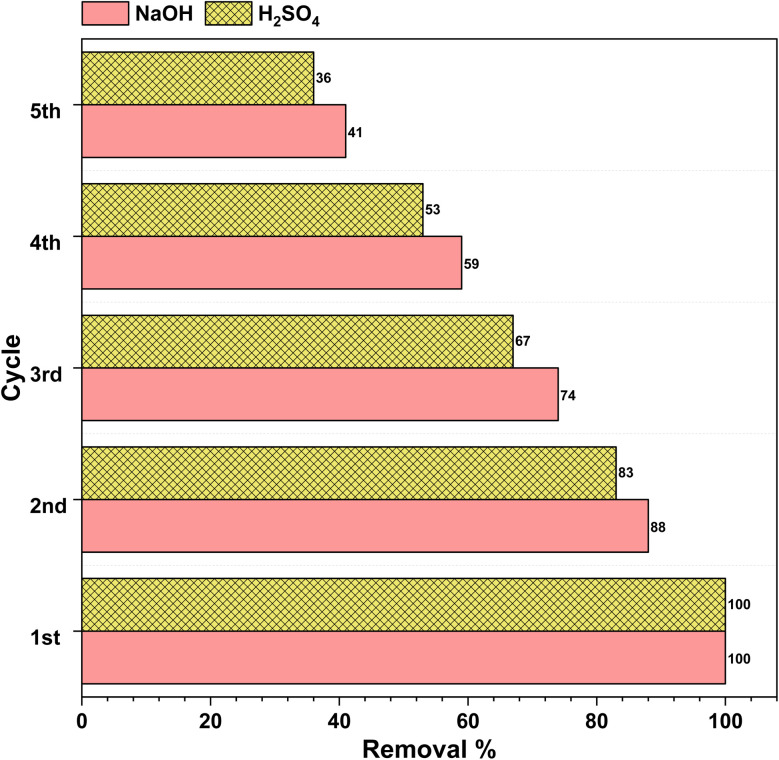
The removal efficiency of the R6G and IC dyes using NaOH and H2SO4 AC after regeneration up to five cycles.

The reusability of the spent adsorbents was carried out by sonicating the spent adsorbent in acetone. Upon sonication the dyes adsorbed onto the adsorbent were dissolved into the solution and free active sites were regenerated which can be used for further dye removal. We observed the efficiency of the adsorbents was decreased with each dye removal step. We could regenerate the adsorbent for five consecutive times but the efficiency of removal for rhodamine 6G and Indigo Carmine was reduced to 41% and 36% of the initial removal capacities for NaOH and H2SO4 AC, respectively, *i.e.*, in the first cycle the adsorption capacities for NaOH and H2SO4 were 751 mg g^−1^ and 226 mg g^−1^, respectively, and in the fifth cycle the adsorption capacities for NaOH and H2SO4 were 307 mg g^−1^ and 81 mg g^−1^, respectively (Fig. 12).

## 4 Conclusion

This study has demonstrated the production of high-performance AC from CS using H_2_SO_4_ and NaOH activation methods. The resulting AC exhibited remarkable dye adsorption capacities, which were further enhanced at elevated temperatures, indicating the endothermic nature of the adsorption process. The comprehensive characterization and isotherm studies confirmed the suitability of the Langmuir model for describing the adsorption behavior, with the AC showing a high affinity for the dye molecules. The investigation into various parameters such as adsorbent dosage, dye concentration, pH, and temperature provided valuable insights into optimizing the adsorption process. The thermodynamic analysis revealed the spontaneous and endothermic characteristics of the adsorption, with negative Gibbs free energy changes confirming the feasibility of the process. This study demonstrates the potential of utilizing CS as a sustainable feedstock for producing efficient adsorbents, contributing to the development of cost-effective and environmentally friendly solutions for dye removal from wastewater.

## Data availability

The original contributions presented in the study are included in the article, further inquiries can be directed to the corresponding author.

## Conflicts of interest

There are no conflicts to declare.

## Supplementary Material

RA-014-D4RA06287F-s001
