# Supplementary material for: High-performance activated carbon from coconut shells for dye removal: study of isotherm and thermodynamics
Source: RSC Adv. 2024 Oct 24;14(46):33797–808. doi: 10.1039/d4ra06287f (PMC11500064; doi:10.1039/d4ra06287f)
Supplement: RA-014-D4RA06287F-s001 [file RA-014-D4RA06287F-s001.pdf]

Supplementary Information (SI) for RSC Advances.
This journal is © The Royal Society of Chemistry 2024

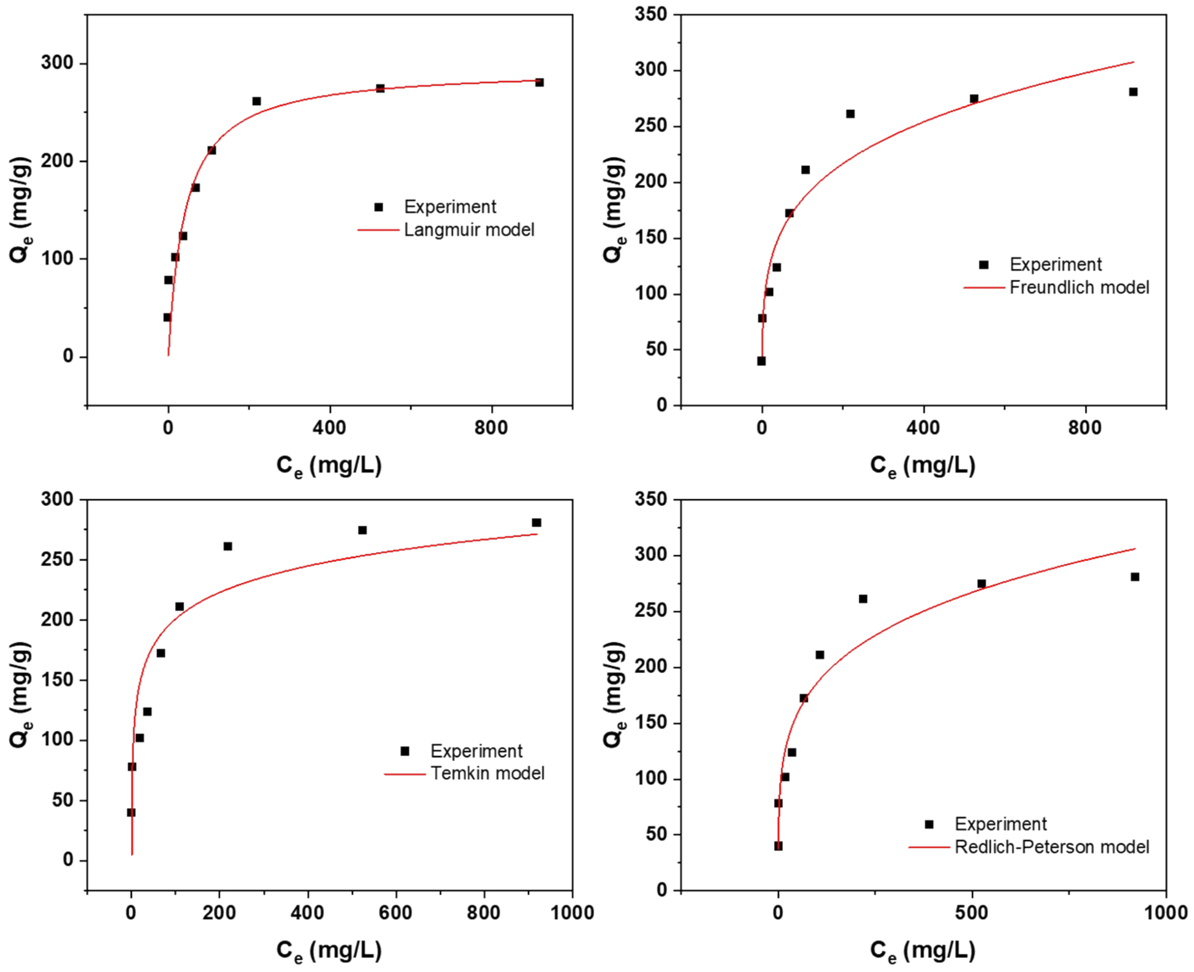

Figure S1: Isotherms for H_2SO_4 activation at 318K

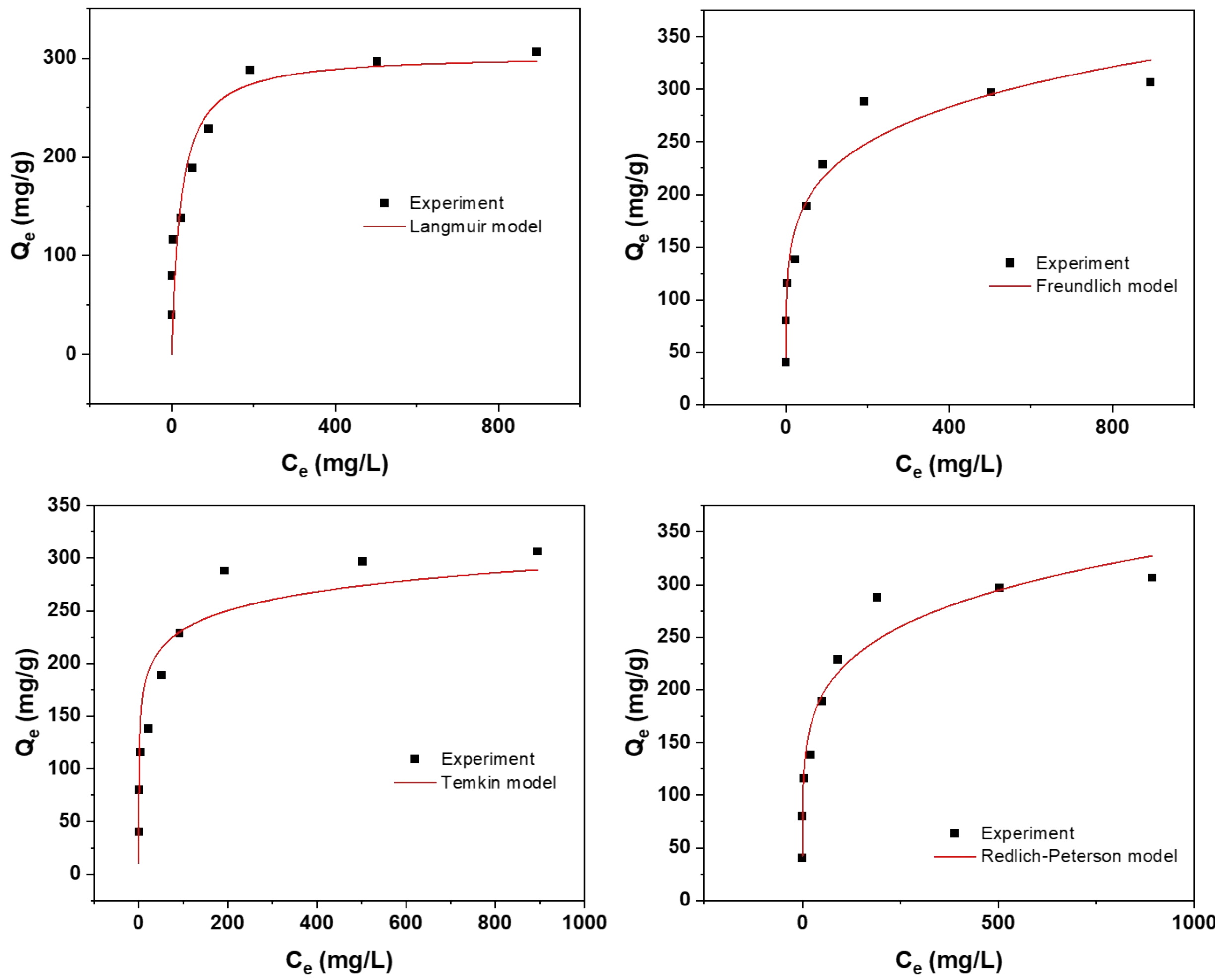

Figure S2: Isotherms for H_2SO_4 activation at 338K

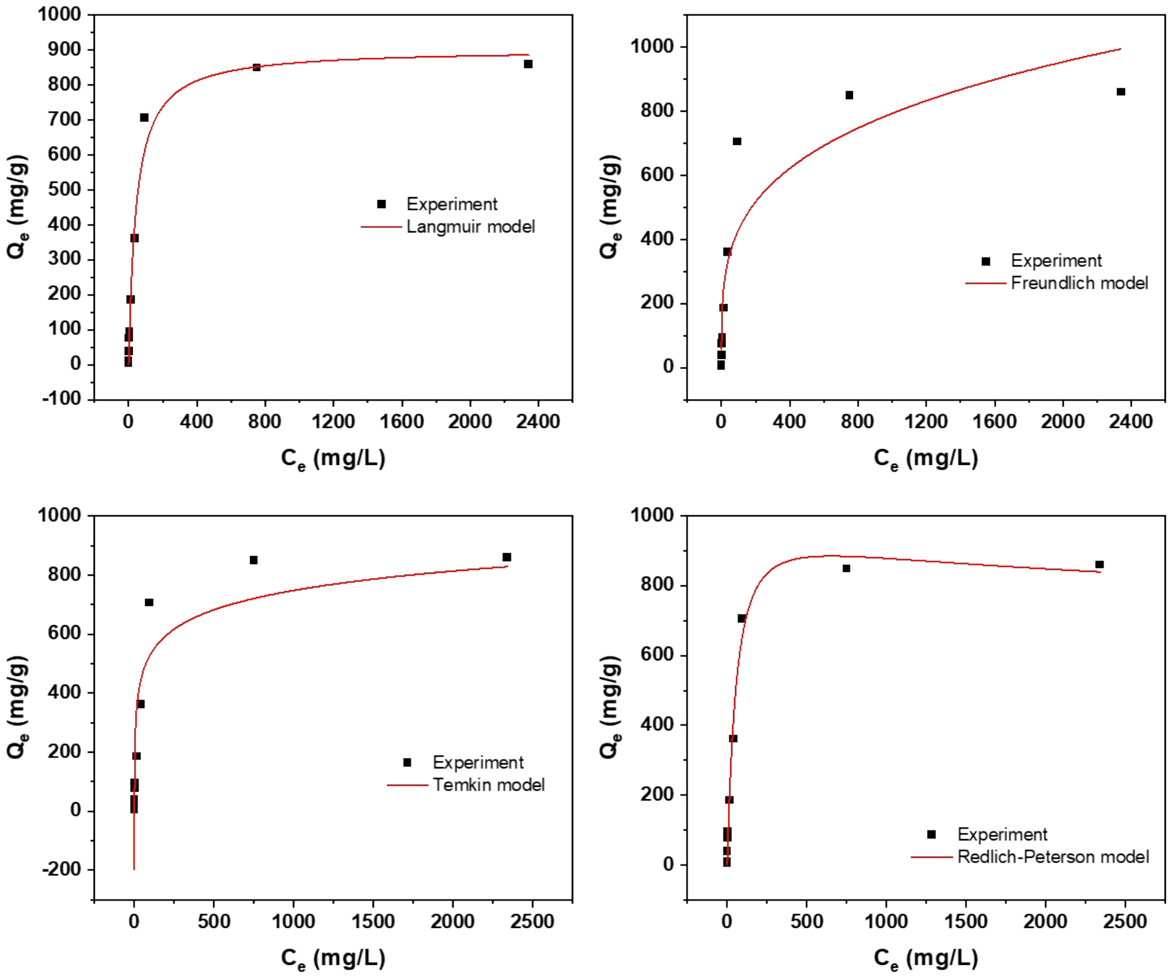

Figure S3: Isotherms for NaOH activation at 318K

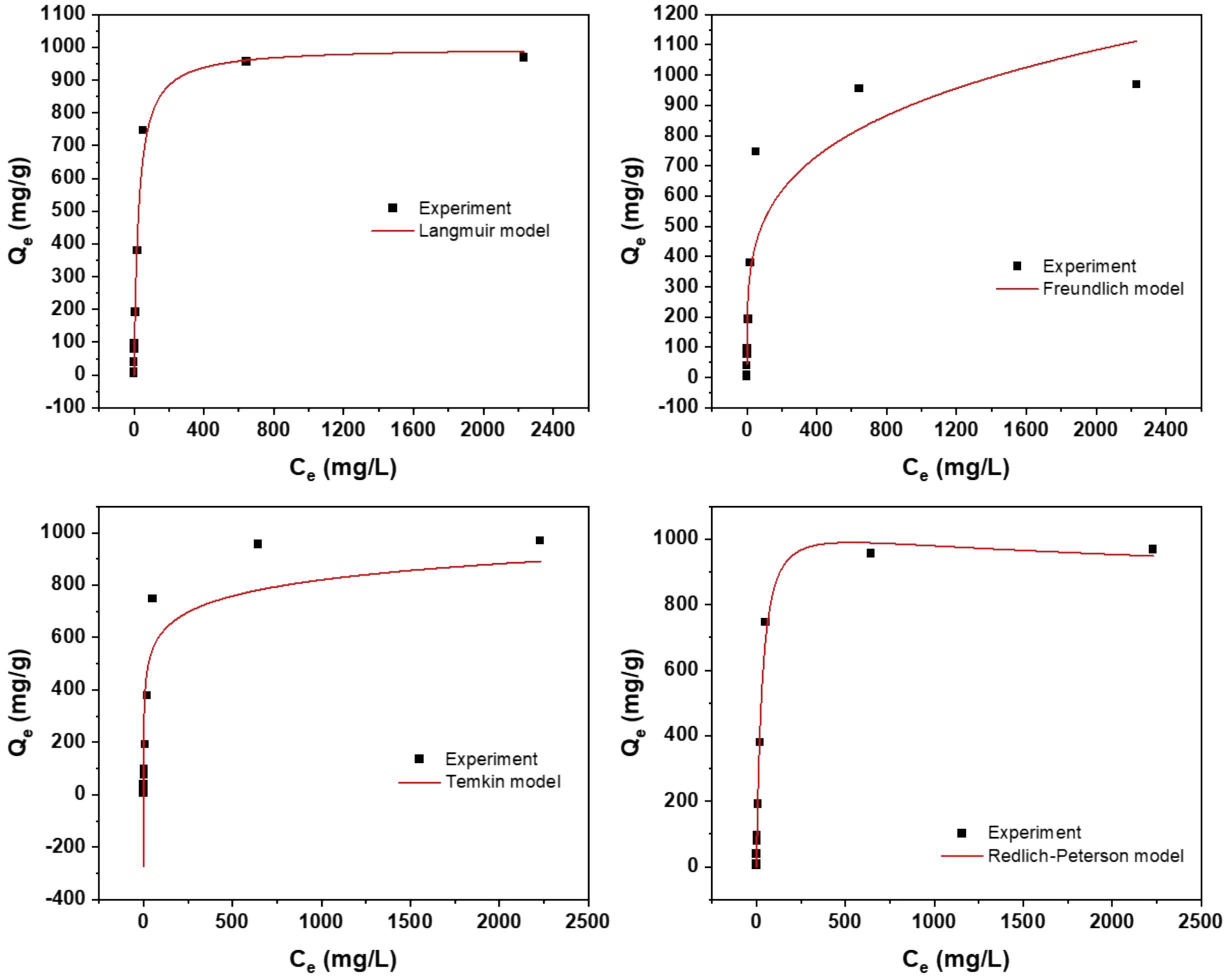

Figure S4: Isotherms for NaOH activation at 338K
